# Supplementary material for: Integrating ecosystem markets to co-ordinate landscape-scale public benefits from nature
Source: PLoS One. 2022 Jan 12;17(1):e0258334. doi: 10.1371/journal.pone.0258334 (PMC8754326; doi:10.1371/journal.pone.0258334)
Supplement: S1 File — (DOCX) [file pone.0258334.s001.docx]

**S1 File**

**Validation and verification of outcomes**

The four peatland schemes and WCC tended to validate and verify outcomes using site visits by independent certification bodies, HB was developing a third-party accreditation system and BIF accredited projects to relevant industry standards. However, validation mechanisms had not yet been developed for NCB and NCPF, and LENs and NIS provided validation in the form of evidence that interventions had been carried out, without requiring independent verification of ecosystem service outcomes (Table 1).

Verified Woodland Carbon Units from WCC projects could be used by companies to compensate for their UK-based greenhouse gas (GHG) emissions but could not be traded on voluntary or compliance carbon markets. A registry enabled units to be bought and sold by companies within the UK. Forward selling of Pending Issuance Units (a promise to deliver GHG benefits on the basis of projections, which cannot be guaranteed or reported as actual GHG benefits) was possible after validation, in addition to the purchase of Verified Carbon Units ex-post. Methods for projecting and verifying carbon sequestration and GHG emission reductions were typically based on or designed to be compatible with methodologies developed for the voluntary carbon market, although none of the schemes currently traded on this market. As a result, GHG benefits were included towards national emissions reduction targets. It was also possible for project developers to make significant savings on verification and auditing compared to the stricter requirements of voluntary market standards like the Verified Carbon Standard. For example, MF and PC allowed the same third-party to carry out both validation and verification and GDNL used a committee of experts to undertake project validation. In the case of the WCC, the methodology for determining carbon sequestration units was initially derived from voluntary carbon market methodologies, and then simplified and adapted to the UK context. Methods for the PC were in turn developed initially on the template of the WCC, but with significant adaptations to enable projects to estimate and then validate GHG emission reductions rather than carbon sequestration. This was done by developing emissions factors based on the analysis of GHG emission data from peatlands of different types and stages of degradation or restoration, in line with the UK’s IPCC Tier 2 methodology used to calculate emissions in the UNFCCC National Inventory Report [59]. Within the PC and the WCC during the development stages and at the start of a project, validation was performed via on-site survey visits to assess the quality and condition of the land and inform the assessment of climate benefits that can be obtained, assess risks to the project and confirm the eligibility of the project.

HB took a similar approach to validation of projects and verification of outcomes, using biodiversity metrics [38,39] to assess changes from a baseline condition assessment on site. Although these were conducted by Environment Bank, there were plans to move to an independent certification body for verification and reporting. The BIF verified project outcomes via an investment committee responsible for assessing the impact of each investment. The basis of these assessments was the Sustainable Blue Economy Finance Principles [60], which were developed by WWF to inform private investment into the blue economy. The Blue Impact Fund’s investments will comply with relevant ASC/MSC standards and Sustainable Development Goals (in particular SDG 14 – life below water). More broadly, verification of outcomes from green finance schemes is being considered by an international Taskforce on Scaling Voluntary Carbon Markets. The Taskforce has suggested in its “initial recommendations”, that satellite imaging, digital sensors and distributed ledger technology would be appropriate technologies to deploy to aid a verifiable and trustable voluntary carbon market with enough integrity for permanence [61].

For LENs and NIS, price negotiations could be based on data regarding the effectiveness of a given intervention and the value of a given service, based on published evidence of the effectiveness of an intervention (sometimes by proxy on the basis that it is included in agri-environmental schemes). Research is being conducted to determine the effectiveness of some LENs interventions via the Resilient Dairy Landscapes project (https://www.resilientdairylandscapes.com/), which may inform future iterations of LENs. LENs also has the ability to ‘adopt in’ measures and outcomes certified under independent standards and codes.

All the peatland schemes defined the GHG result as avoided emissions resulting from the (partial) rewetting of drained peatland and integrate both CO₂ and CH_4_ using CO_2_eq/year. PC, MF and GDNL all used emissions factors to estimate GHG emission reductions after restoration, based on functional relationships between mean annual water table depth, vegetation composition and GHG fluxes [62,63,64]^,^. MF was the first scheme to apply site-specific emission factors (Greenhouse gas Emission Site Types, GEST) to rewetted peatlands. Soon after, the Verified Carbon Standard incorporated this approach into its methodology for rewetting of temperate peatlands on the voluntary carbon market [65]. The approach developed for PC applies the same logic and defines five condition categories based on land degradation and vegetation characteristics. The emissions factors used by the different schemes are not directly comparable as all schemes have stratified eligible peatlands into different condition categories, and then calculated emissions factors for each of these categories. Moreover, the functional relationship underlying vegetation-based GHG emission site types varies with biogeographic region and has to be calibrated for other biogeographical and climatic zones [66]. Experienced surveyors can classify degraded and drained peatlands from field visits, which can be converted to GHG using emission factors in look-up tables. GDNL and PC are the only schemes to use emissions factors for partial rewetting where the land use is not changed, and MF is the only scheme to offer emissions factors for rewetting combined with a land use change to paludiculture. This approach requires sufficient evidence of GHG emissions for different degradation states and peat and vegetation types, restricting the range of peatlands that can be included in schemes (the PC is currently restricted to blanket bogs on this basis). There was insufficient published evidence to construct emissions factors for MM, so it relies instead on an annual peat decomposition rate of 1 cm per year, which it is assumed can be halted by rewetting.

**Additionality and leakage**

Additionality was only assessed formally by the four peatland schemes, WCC and HB, typically via legal (e.g. projects go beyond what would be required by law), financial (e.g. projects would not be possible without carbon finance) and other additionality tests (e.g. application of biodiversity metrics in HB receptor sites). None of the other schemes applied formal additionality tests, relying instead on trusted intermediaries to manage additionality informally as part of the project design process (e.g. LENs) or identifying businesses that had been unable to fund sustainability initiatives via other means (e.g. BIF) (Table 1).

The principle of additionality is an important requirement for ecosystem markets, ensuring that benefits arising from projects would not have occurred without investment, and would not have occurred anyway without it [67]. However, assessing additionality is complex and represents a procedural hurdle to project development. Although the PC allowed for group certification for small projects to lower costs associated with proving additionality, interviewees suggested that additionality test could be simplified or externally supported across all the schemes that included formal additionality tests. These schemes typically applied legal and financial tests. Financial additionality requires that a project would not be economically feasible without carbon finance. PC, WCC, MF and MM all used different methods to test for financial additionality, while the PC, WCC and GDNL also accounted for legal additionality, requiring projects to produce emission mitigation beyond what would be achieved by activities or interventions already required by policy and regulation. PC, WCC and MM allowed public and private finance to be combined and considered projects to be additional as long as 15%, 15% and 10% of project costs were from carbon finance respectively. However, MF explicitly excluded the use of public funding in projects. PC and WCC also included barrier tests, enabling a project to be additional if it is unable to meet the financial additionality test if it has overcome other barriers that would have otherwise prevented peatland restoration.

LENs and NIS did not have defined measures for ensuring additionality, with one of the interview respondent for LENs arguing that:

*“Additionality is less of a concern with landscape outcomes in which the purchaser has a direct technical interest. An example would be businesses interested in reducing their exposure to flood risk or costs relating to water quality, or consortia of businesses and local government interested in regional ‘placemaking’. In these instances, the purchaser has a direct interest in the technical outcome of the work they are paying for, and less interest in the attribution of payments. It follows also that if the outcome is already being delivered, then the market for it – the incentive to pay – disappears. The more important test here is quality assurance for the buyer, and the main challenge faced may be that of ‘free riders’ – i.e. beneficiaries of landscape outcomes who do not pay.”*

(Scheme representative, Landscape Enterprise Networks)

Leakage (where damaging activities are displaced to another location by a scheme) was implicitly avoided in LENs projects due to the landscape scale at which they operate, reducing the likelihood that damaging activities are displaced to neighbouring land. The MF provided guidelines on how to minimise leakage by site selection or the provision of alternative income sources to avoid leakage via activity shifting. In the WCC and PC, leakage had to be included in net GHG emission savings estimates for the project. The MM standard concentrated their GHG accounting on degraded peatlands, which were no longer in agricultural usage (so there was no agricultural activity to displace). HB did not allow existing high-quality habitats to be converted into other habitats, but it did not assess whether landowners brought high quality habitats that were not covered by the scheme into production to compensate for reduced production. Leakage was not explicitly considered in the other schemes reviewed.

**Permanence**

Permanence was addressed primarily via contractual arrangements in the schemes reviewed, although Conservation Burdens (Scotland) and Covenants (England and Wales) were sometimes proposed by schemes as potential future options to provide additional assurances to buyers in some UK schemes, and BIF provided follow-on funding opportunities to enhance permanence.

The permanence of funded land use changes and other interventions, and hence outcomes of schemes was not formally assessed for NIS or GDNL projects, and was provided via contractual agreements for LENs and MM projects with no protection against reversals outside contract periods. However, in the case of LENs, three measures were taken to increase longevity beyond contractual obligations: (1) selection of measures that are hard to reverse (e.g. capital works), (2) long-term payments to maintain management measures, and (3) selection of multifunctional measures, with multiple rationales and revenue streams associated with maintaining the measure. In the case of MM, the likelihood of reversals was implicitly limited by site selection, focusing on land that had been taken out of agricultural production. The absence of guarantees over the permanence of interventions and associated outcomes did not appear to be a major barrier to investment in these schemes.

The only schemes that provided legal protections around permanence were MF and WCC, which exploited existing national/regional laws or procedures allowing to prescribe water levels (MF) or preventing deforestation (WCC). MF guarantees permanence by ensuring the administrative and legal basis of the project planning and approval process and by securing the permanent availability of the project area, either through acquisition of the land or through registration of servitude with respect to the water table in the land register. In the absence of legal protection, the PC relied primarily on contractual agreements, but was able to provide further assurance via conservation covenants in England and Wales and conservation burdens in Scotland (some WCC projects had also used these). Conservation covenants are private and voluntary agreements between landowners and responsible body (e.g. local authority) who is responsible for monitoring and enforcing the obligations of the covenant. Conservation burdens were established under the Title Conditions (Scotland) Act 2003. The Act codifies the kinds of restrictions or burdens that can be included in titles to land and property, and establishes conservation burdens as a voluntary instrument that can be used to protect, enhance and maintain aspects of natural and cultural heritage. Covenants and burdens can only currently be agreed by “responsible bodies” who hold the covenant on behalf of the public. In Scotland there are a number of conservation charities and public bodies who hold this status, but in England the National Trust is currently the only body with formal covenanting power (Under National Trust Act 1937, s.8). This will change with the implementation of Part VII of the Environment Bill 2020, which will empower a range of public and charitable bodies to hold the benefit of a covenant as “responsible” bodies. The Bill, when passed into law, will also enable a wider range of ecosystem service buyers to use them to create long term “property” interests (rather than shorter term contracts) under the WCC and PC [68,69].

Under the terms of a mandated regime for biodiversity net gain it is a requirement for the land supplied to generate credits – i.e. habitat banks and bespoke offset sites – to be subject to a 30 year agreement with the payer of the credits. The Environment Bank’s HB model involves the issuance of a Conservation Bank Agreement between Environment Bank and the landowner provider which has a term of 30 years. The conservation credits generated by the HB or offset site are sold to the developer under a Conservation Credit Purchase Agreement. The funds generated are used to pay the landowner a) capital costs of the establishment of the specific habitat or habitat mosaic and b) annual management costs with RPI and some income foregone/profit. Environment Bank holds the funds and pays out according to an agreed payment schedule following monitoring visits to ensure that the project is delivering against milestones set out in a Biodiversity Management Plan that accompanies the Conservation Bank Agreement. It is considered that a 30-year term offers a good compromise between encouraging landowner participation and delivering habitat creation and restoration at scale. By year 30 one would expect the majority of habitats to be reaching some form of maturity (woodland excepted). It is highly unlikely that after 30 years a landowner, if selected correctly in the first place, would wish to impact on the landscape and biodiversity enhancements that a habitat bank would deliver to his/her land by its removal and conversion back to agricultural production. In the unlikely event, however, that there were imperative reasons for needing the land for development, the landowner would need to pay the significant expense of offsetting the impacts of that development. Within 30-years it is likely, in any event, that planning policy will exclude the use of offset sites and habitat banks for development as part of future planning policy compliance.

**Supply and demand issues**

All schemes were voluntary, open marketplaces and the level of compensation for interventions was primarily determined by project costs, with negotiation between suppliers and buyers possible in some schemes. None based their prices on the price per tonne on the voluntary carbon market, which would typically have been too low to cover project costs. One of the ways that projects justified higher prices (compared to carbon market prices) was by highlighting additional non-carbon benefits. Most schemes used intermediaries to engage with project developers (e.g. landowners and tenants), or the scheme itself performed this function (e.g. BIF) and LENs used supply aggregators to aggregate sufficient density of supply within a single landscape. However, engagement with suppliers (typically landowners and managers) was a challenge for all schemes except BIF which had created a £90M project portfolio prior to entering its investment phase (Table 1).

Four of the schemes (LENs, NIS, WCC and PC) enabled negotiation between buyers and sellers through intermediaries (a single intermediary worked on behalf of LENs and NIS, and multiple independent intermediaries facilitated investments in WCC and PC). WCC determined price on project-by-project basis between suppliers and buyers (except for the Woodland Carbon Guarantee in England, whereby the government offers a contract for the option to sell Woodland Carbon Units to them *via* an auction). Similarly, the PC negotiated prices per project primarily on the basis of costs, which could vary considerably between projects, based on accessibility, level of degradation and other factors. LENs and NIS used a “supply aggregator” (typically a locally trusted organisation contracted to engage landowners/tenants across a landscape in the scheme) as a broker on behalf of the sellers to negotiate deals with demand side actors for the delivery of interventions. Prices were negotiated as a bundle of ecosystem services typically including soil function, water quality and biodiversity with limited quantification of likely risks or benefits. In contrast, negotiation was not possible in MF and MM projects, which were based on cost alone, with fixed prices for buyers. Prices for MF credits are based on the costs of their production, i.e. calculated by deriving the costs of implementation, divided by the total amount of emission reduction units for sale over the project crediting period.

Compensation mechanisms varied significantly across schemes (and in some cases between interventions within schemes) with the use of different legal agreements and payment structures. For example, PC projects may be paid outright by a buyer or by an intermediary who then sells carbon units to multiple buyers, with payments structured in a single on-off payment for the whole project, or with maintenance and revenue payments annualised after an initial lump sum to pay for capital works. All payments go via MF as the issuer and broker of uniquely identified credits, whereas MM and PC do not handle transactions, which take place directly between buyers and sellers, often via intermediaries. GDNL uses a central registry and issuer to keep track of uniquely identified credits which can be traded between buyers, to allow for aggregation and selling of credits from many different sectors alongside peatland restoration.

LENs is currently developing the legal framework to support its delivery at scale. It currently operates on the basis that a group of demand-side players come together to agree to co-procure (via a “demand aggregator”) a certain proportion of ecosystem services or other outcomes and form a memorandum of understanding to achieve this. At this stage this is not a contract. It is envisaged, however, that in due course LENs arrangements will mature in a process leading to the conclusion of multiple linked contracts between demand side aggregators and supply side aggregators/actors (for example farmers and other land managers delivering ecosystem services). LENs suggest that in the future a regional entity could be established to centrally manage multiple contracts. The legal delivery mechanisms for NIS are under development. Although similar to LENs, they identify a role for a centralised entity to manage contractual arrangements. There was also a recognition that contacts were required to be robust, whilst also being flexible, particularly in the case of long-term landscape interventions where there may be requirements for suppliers and/or the interventions to change over time, for example, in the case of flood risk management to accommodate emerging climate change challenges.

Engagement with project developers (often landowners) was a challenge for many schemes. The new General Data Protection Regulation (GDPR) regulation in Europe made it difficult to contact landowners at scale, requiring alternative, often resource intensive means of communication e.g. via trusted brokers. Landowners were not generally willing to self-organise or cooperate with others to develop projects or other proposals for buyers, relying instead on intermediaries to support collaboration and represent their collective interests. There was also a reluctance among the landowning community to enter into long-term agreements. This was particularly problematic for schemes such as the WCC and PC which required permanent commitments for at least 30 years. Moreover, there were concerns from landowners that peatland restoration under the PC could lead to areas of wetland and scrub that would: i) not be eligible for agri-environment payments; ii) not be eligible for Agricultural Property Relief or Business Property Relief, increasing liabilities under Inheritance Tax law; and iii) lead to designation of Sites of Special Scientific Interest, leading to increased statutory obligations and commitments on the land [42]. Similar concerns were expressed by landowners engaging with MF in Germany.

On the demand side, many potential investors were unwilling to share commercially sensitive data to enable the establishment of consortia, making it difficult to establish co-procurement arrangements. For schemes that lacked formal additionality criteria (see section 3.2), investors were reluctant to pay for interventions that they perceived farmers or landowners should be doing as part of compliance with regulation and/or that could be paid for by public finance.

Across the schemes, specific integration and consideration of the wider social distribution of ecosystem services was limited, although there was recognition of its importance for buyers with Corporate Social Responsibility goals. Interviews with businesses during the pilot phase of the PC suggested that companies may be willing to pay a premium for peatland carbon on the basis of project location and co-benefits, with water and biodiversity co-benefits of particular interest [69]. The PC only stipulates the need for a statement of environment in the management plans of projects, which can include the delivery of additional ecosystem services. In contrast, MF provides detailed methodologies for quantifying co-benefits covering improved water, food mitigation, increased groundwater store, evaporative cooling, and increased mire-typical biodiversity [66,67]. MF also makes project areas accessible for buyers to visit, in contrast to more ‘anonymous’ overseas carbon projects. The WCC adopts a ‘Wider Benefits Tool’ to provide a consistent way of evaluating the likely benefits of woodland creation in relation to four aspects; *water, community, biodiversity* and the *economy*. While the tool does not quantify the benefits delivered, it is a consistent way of evaluating the likely benefits and relative merits of each project. By driving multiple investments in multiple landscape functions, LENs seeks to make landscapes more responsive to societal needs, however, the emergent outcomes are not currently measured. Despite limited quantification of wider benefits across the schemes, the potential to further integrate co-benefits was recognised, as long as monitoring costs were not prohibitive.

In relation to HB, prices of conservation credits are currently set by Environment Bank in their model based on known costs of establishment and 30-year management. Credit pricing is positioned in terms of what the market will demand or bear, which varies in terms of location, geology/geography, habitat type, hydrology, soils etc. Planning Authorities are key to ensuring demand by delivering on their (to be) mandated duty to require development to deliver at least 10% Biodiversity Net Gain. Having a mandated system is likely to provide clarity and certainty to developers and reduce planning delays. Supply of sites for HB is limited by a number of factors, including concerns from landowners around inheritance tax rules, contract lengths, risks of land being designated a protected sites for conservation and concerns about engaging with environmental NGOs who have insufficient access to land in the appropriate places. Planning Authorities may use their own land but that is likely to be legally challenged in the future because, in governance terms, regulators must not financially benefit from those whom they regulate.

Regional ecosystem markets share elements with club goods [53,54]; that of being excludable, with reduced rivalry until specific forms of “congestion” caused by a high number of buyers appear. Excludability is provided by the fact that participation to the schemes is necessary to correct market distortions that do not spontaneously generate the right amount of public services. Rivalry is also possible, but not related to the disutility caused by “congestion”. Benefits received by a buyer of ecosystem services do not constrain the ability of others to get the same amount of environmental services. From this perspective, each individual’s consumption of public goods leads to no subtraction from any other individual’s consumption of that good [55]. However, rivalry could be configured as “saturation in marginal benefits” beyond a certain threshold of investment after which any marginal change in the benefits provided by the provision of ecosystem services is not perceived by the investors. Thus, buyers reaching the threshold may perceive that the cost of participating in the schemes may be too high compared to the marginal value received, with the consequence of generating a form of disutility (under this perspective the schemes can be configured as a club good). If scheme participants understate the public benefits received, they reduce the effort they supply towards achieving the club's collective goals and take advantage of other club members by free-riding.

In addition, the use of fixed costs (as it happens in schemes like Moor Future and Max Moor) rather than negotiated prices between buyers and sellers may limit the optimal provision of public goods. This happens because price negotiation should generate efficient participation in schemes allowing buyers to subscribe until marginal benefits from consumption equalize “congestion” costs. If costs are fixed, this mechanism does not work. A correction can be made by imposing a two-part pricing approach: one is the fixed up-front fee to participate; and the other is the per unit charge to achieve an optimal number of participants. This approach could be also adopted by those schemes that are partially supported by public funds, with Government supplying a fixed up-front fee, and private investors negotiating the delivery of ecosystem services across the investor community in order to reduce free-riding.

**Interaction with public funding**

Schemes relied to varying extents on public funding, both in terms of scheme operation and project financing. The peatland schemes (with the exception of MF) and WCC were significantly more reliant on public funding for project financing and in many cases scheme operation than the other schemes reviewed (Table 1).

LENs, NIS, MF and the green finance schemes were least dependent on public funding for project financing. MF projects had to be fully funded through private carbon finance. LENs projects were financed completely by private finance, and the majority of LENs farmers interviewed by Coyne et al. [56] were not engaged in publicly funded agri-environment schemes. It is not clear to what extent landowners engaging in NIS would supplement scheme payments with public funding via agri-environment schemes, but the scheme was not designed to rely on substantial cross-subsidy from Government. A number of the green finance schemes were being developed in collaboration with Government agencies, as part of a wider route map developed by the Scottish Conservation Finance Project, led by Scottish Environment Protection Agency and the Scottish Wildlife Trust. As a result, each of these relied on a small amount of public funding indirectly via grant-based research funding and in-kind support to develop the pathways to finance. WCC, PC, MM and GDNL were more dependent on public funding for scheme development and operation, and these schemes also relied heavily on public funding for projects, with private contributions only having to account for 10-15% of project costs. In some cases, public funding was used to pay for intermediaries, supply aggregators and other advisors working with landowners. MM is a public-private partnership and bases project implementation on public co-funding with a share of up to 90%, while the remainder of the investment needs come from carbon finance.

Where relevant, this reliance on public funding was a major source of uncertainty for schemes, as changing priorities and lack of public funding in the aftermath of the financial crisis meant that several projects could not get funding for already planned activities. As a result, some schemes also drew on charitable sources to support their activities. There were also concerns about the design of public funding for woodland creation and peatland restoration in the UK. One interviewee explained how the high prices achieved under Woodland Carbon Guarantee auctions had undermined demand for woodland projects being sold by Forest Carbon Ltd. While this interviewee suggested that the design of public schemes should be altered to avoid competition with the private sector, another interviewee suggested that such auctions should drive alternative, more cost-effective and competitive private investment models for financing woodland creation. Similarly, there were reports of NatureScot’s Peatland Action programme crowding out Peatland Code projects, given the relative simplicity of accepting funding for restoration with the aid of Peatland Action facilitators with more familiar, shorter contracts. Given the scale of public spending planned for natural capital investment in the UK, with more than £90 million for the Nature for Climate Fund and an additional £40 million investment in nature recovery through Green Recovery Challenge Fund, these concerns are only becoming more acute.

At present HB, whilst already scaling, has not attracted Government direct funding, but Biodiversity Net Gain is soon to be mandated in the English planning system and is being considered elsewhere in the UK, which is likely to drive demand. Although HB is already offered by a private sector broker, the UK Government is planning to set up its own biodiversity credits scheme and sales platform for developers who cannot find a local market, which one interviewee suggested could inadvertently create competition between public and private HB providers.

References

57. Brown P, Broomfield M, Cardenas L, Choudrie S, Kilroy E, Jones L, MacCarthy J, Passant N, Thistlethwaite G, Thomson A, Wakeling D. UK Greenhouse Gas Inventory, 1990 to 2016. Annual Report for Submission under the Framework Convention on Climate Change. 2018, HMSO, London.

58. European Commission. Introducing the Sustainable Blue Economic Financing Principles. 2018. https://ec.europa.eu/maritimeaffairs/sites/ maritimeaffairs/files/introducing-sustainable-blue-economy-financeprinciples_en.pdf

59. TSVCM (Taskforce on Scaling Voluntary Carbon Markets). Taskforce on Scaling Voluntary Carbon Markets: Consultation Document. 2020. https://www.iif.com/Portals/1/Files/TSVCM_Consultation_Document.pdf

60. Couwenberg J, Thiele A, Tanneberger F, Augustin J, Bärisch S, Dubovik D, Liashchynskaya N, Michaelis D, Minke M, Skuratovich A, Joosten H. Assessing greenhouse gas emissions from peatlands using vegetation as a proxy. Hydrobiologia. 2011, 674: 67-89.

61. Joosten H, Brust K, Couwenberg J, Gerner A, Holsten B, Permien T, Schäfer A, Tanneberger F, Trepel M, Wahren A. MoorFutures: Integration of additional ecosystem services (including biodiversity) into carbon credits – standard, methodology and transferability to other regions. BfN-Skripten 207. Federal Agency for Nature Conservation, 2015. https://www.bfn.de/fileadmin/BfN/service/Dokumente/skripten/Skript407.pdf

62. Tiemeyer B, Freibauer A, Borraz EA, Augustin J, Bechtold M, Beetz S, Beyer C, Ebli M, Eickenscheidt T, Fiedler S, Förster C. A new methodology for organic soils in national greenhouse gas inventories: Data synthesis, derivation and application. Ecological Indicators. 2020, 109: 105838.

63. VCS (Verified Carbon Standard). VM0036 Methodology for Rewetting Drained Temperate Peatlands. v1.0, 2020. https://verra.org/methodology/vm0036-methodology-for-rewetting-drained-temperate-peatlands-v1-0/ (accessed: December 2020).

64. Rodgers C. Delivering a better natural environment? The Agriculture Bill and future agri-environment policy. Environmental Law Review. 2019, 21: 38-49.

65. Rodgers C, Grinlinton D. Covenanting for Nature: A Comparative Study of the Utility and Potential of Conservation Covenants. Modern Law Review. 2020, 83: 373-405

66. Bonn A, Reed MS, Evans C, Joosten H, Bain C, Farmer J, Emmer I, Couwenberg J, Moxey A, Artz R, Tanneberger F, von Unger M, Smyth MA, Birnie R. Investing in nature: developing ecosystem service markets for peatland restoration. Ecosystem Services 2014, 9: 54-65.

67. Von Unger M, Emmer I, Joosten H, Couwenberg J. Design an International Peatland Carbon Standard: Criteria, Best Practices and Opportunities (Final Report). German Environmental Agency. Climate Change 42/2019. 2019. https://www.umweltbundesamt.de/sites/default/files/medien/1410/publikationen/2019-11-28_cc-42-2019_sca_peatland_standards_0.pdf

68. Buchanan J., 1965. "An Economic Theory of Clubs," Economica, 32(125), N.S., pp. 1-14.

69. Richard Cornes, Todd Sandler (1996) "The Theory of Externalities, Public Goods and Club Goods", in Cambridge University Press, 2nd ed., pp. 347-356.

70. Samuelson, P. A. (1954). The Pure Theory of Public Expenditure. The Review of Economics and Statistics, Volume 36, Issue 4 (Nov., 1954), 387-389.
